# Brazilian tracheotomy speech valve: diaphragm pressure standardization

**DOI:** 10.1016/S1808-8694(15)30840-5

**Published:** 2015-10-18

**Authors:** Ângela Rúbia Oliveira Silveira, Marcelo Naoki Soki, Carlos Takahiro Chone, Ronny Tah Y Ng, Eduardo George B. Carvalho, Agrício Nubiato Crespo

**Affiliations:** 1 Physician, 2nd year resident, Otorhinolaryngology and Head & Neck Discipline, Faculdade de Ciências Médicas, Universidade Estadual de Campinas, UNICAMP.; 2 Physician, 2nd year resident, Otorhinolaryngology and Head & Neck Discipline, Faculdade de Ciências Médicas, Universidade Estadual de Campinas, UNICAMP.; 3 Adjunct professor, Coordinator of the Head & Neck Unit, Otorhinolaryngology and Head & Neck Discipline, Faculdade de Ciências Médicas, Universidade Estadual de Campinas, UNICAMP.; 4 Physician, 4th year resident, Otorhinolaryngology and Head & Neck Discipline, Faculdade de Ciências Médicas, Universidade Estadual de Campinas, UNICAMP.; 5 Physician, assistant physician in the Otorhinolaryngology and Head & Neck Discipline, Faculdade de Ciências Médicas, Universidade Estadual de Campinas, UNICAMP.; 6 Adjunct professor, head of the Otorhinolaryngology and Head & Neck Discipline, Faculdade de Ciências Médicas, Universidade Estadual de Campinas, UNICAMP.

**Keywords:** airway obstruction, rehabilitation, tracheotomy

## Abstract

Tracheotomy is performed in cases of upper airway obstruction or chronic pulmonary disorders. The Tracheotomy Speech Valves (TSV) improve communication and airway hygiene and humidification of tracheotomized patients. **Aim:** To show the low cost Brazilian TSV and its use in speech rehabilitation of tracheotomized patients, to evaluate diaphragm opening resistance and comfort to the patient. Study Design: Experimental, contemporary cohort. **Materials and methods:** The TSV was used in 32 patients. The valve has a diaphragm within a stainless steel body with plastic fittings. We studied the level of respiratory comfort according to the degree of valve diaphragm resistance, 40, 50 and 60 shores. **Results:** All the patients used the TSV coupled to the cannula in a regular basis, 26 of them did it for more than 12 hours daily and from these, 14 used it for 24h daily. The diaphragm pressure obtained was that of 40 shores for 13 patients and 50 shores for 19 patients. 60 shores was never used. **Conclusion:** the metal TSV helps with speech without the need for closing the cannula with one’s finger, and breathing was comfortable. We achieved standard diaphragm resistance. Currently all the patients from this study use this TSV with speech and 43.75% use it full time.

## INTRODUCTION

Tracheostomy is a procedure that is done when there is upper respiratory obstruction or chronic lung disease, to facilitate lung hygiene and to reduce the dead space.

One of the most significant issues in tracheostomy is loss of verbal communication or inadequate development of voice communication in children.^1^, ^2^, ^3^, ^4^, ^5^, ^6^ In this case, verbal communication is critical in the patient’s global care, psychological status and social interaction.^4^ In children with tracheotomies,^5^ especially from five to nine months of age, the development of communication (receptive and expressive language) may be compromised.^2^ Other functions of the upper airways may be compromised in patients with tracheotomies, including: warming, humidifying and filtering the air, coughing, sneezing, taste, smell, and swallowing.^3^ Regarding the latter, an increased rate of aspiration has been demonstrated in tracheostomized patients.^6^, ^7^, ^8^, ^9^, ^10^, ^11^, ^12^, ^13^ Elevation of the larynx is also affected since the cannula fixates the larynx to the skin of the neck.^14^

Speaking valves (VF) for tracheostomy have minimized these procedure-associated losses. There are the Passy-Muir, Montgomery, Olympic and Kistner valves, of which the Passy-Muir valve provides the best voice quality, as verified by listeners and patients;^4^ it also has the lowest rate of mechanical problems.^4^ Speaking valves are unidirectional and allow air to enter with little inspiratory pressure while inhaling. The valve closes during phonation and air is directed to the larynx. All valves in this country are imported, which increases the cost for patients, a cost not covered by the Unified Health System (Sistema Unico de Saude) or medical insurance companies. Thus, a Brazilian valve was developed to fill in this gap and to improve the quality of tracheostomized at a lower cost. There was no standardized diaphragm hardness to make it feasible to sell the diaphragm with the adequate opening pressure, without the need for individual adjustments.^15^ The purpose of this study was to present a fully Brazilian tracheostomy speaking valve - in which the diaphragm hardness was standardized - which was used in 32 patients; an assessment was made of the degree of respiratory comfort according to the valve opening resistance.

## MATERIAL AND METHOD

A contemporary cohort study was undertaken. All patients in this study signed a free informed consent form. The Research Ethics Committee approved this study (number 0715.0.146.000-07, document number 975/2007).

Thirty-two tracheostomized patients with metallic cannulae were selected for using the speaking valve. The age of patients ranged from 5 to 58 years (mean 46 years). Indications for tracheostomy were partial laryngectomy in 26 patients (laryngeal/pharyngeal tumors) with no tracheoesophageal fistulae, long-term orotracheal intubation in 4 patients, and bilateral vocal fold paralysis in 2 patients. All patients were monitored weekly during the first month, and then 60 and 90 days after placing the speaking valve. Patients with laryngotracheal stenosis above the tracheostomy requiring that the tracheostomy cannula balloon remained insufflated permanently were excluded.

**Figure 1:**
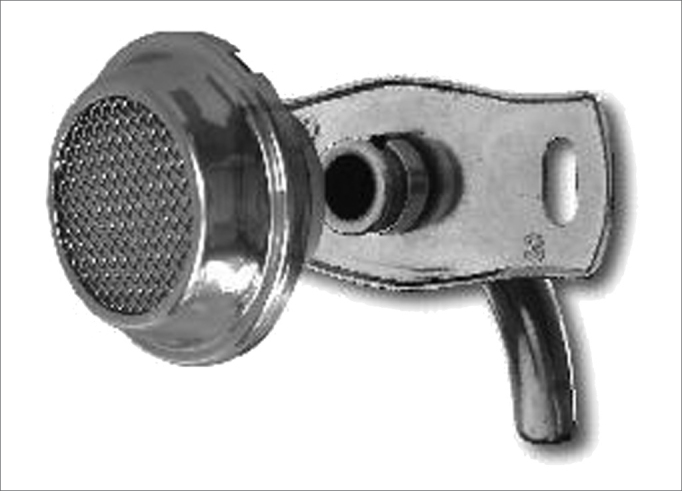
Speaking valve coupled to the metal cannula.

**Figure 2:**
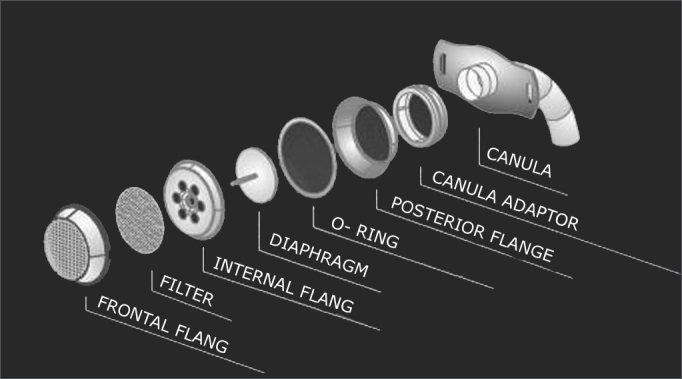
Diaphragm, filter and connections.

The speaking valve developed for this study contains a diaphragm, a filter and plastic connections mounted within a stainless steel body (Fig. 1). It may be adapted to all Brazilian metal cannulae (Fig. 2) by an anterograde route on a dock in the speaking valve adaptor connected to the headpiece of the internal tracheostomy cannula. It has an adaptor for all tracheostomy cannulae numbers. Forty, 50 and 50 Shore (valve opening hardness) diaphragms were tested; the 40 Shore diaphragm offers the least resistance to opening. All patients started with 50 Shore valves; after the first week, and according to the difficulty in inhaling, patients could change to 40 Shore diaphragms; otherwise if air was escaping, a 60 Shore diaphragm was placed. All patients were assessed weekly during the first month to assess the comfort of using the valve and to set the best valve hardness for each patient; this was done using a specific questionnaire to verify speaking without occlusion of the cannula, effort in speaking, the practice of physical exercises, the frequency at which the cannula had to be cleaned, coughing, sleeping with the cannula, speaking in public and duration of daily use.

## RESULTS

All 32 patients adapted to the speaking valve, presenting adequate and effortless speech. Sports activities, walking with effort and speaking with no need for digital occlusion of the cannula were possible in all patients. There was less tracheal secretion issued through the cannula; all patients reported improved local hygiene, especially about coughing and digital occlusion in public places. The indications for speaking valves are the same as those for patients using tracheostomy metallic cannulae.

All patients adapted well to the tracheostomy cannula valve and found it easy to handle. There were no reports of accumulated secretions, oxidation of materials or breathing difficulties. One patient only reported that the valve had to be removed urgently because the diaphragm locked.

The daily duration of valve use was as follows: 14 patients used it the whole day, 11 patients used it during 16 hours, 1 patient used it during 14 hours and 5 patients used it during 10 hours (Table 1).

**Table 1:** Duration of daily use of the valve in hours X the number of patients

	Duration of use in hours (h)				
	10	14	16	24	no recording
Number of patients	5	1	11	14	1

No patient required changing to a 60 Shore valve; after one week of valve use, 13 patients chose to change to the 40 Shore valve to increase their inspiratory comfort (Table 2).

**Table 2:** Valve diaphragm resistance in Shores X the number of patients

	Resistance of the diaphragm		
Number of patients	40	50	60
	13	19	0

## DISCUSSION

Since 1975 techniques have been developed to make it possible for tracheostomized patients to speak.^12^, ^16^, ^17^ Speaking valves may be used in the neonatal period at a minimum age of 13 days.^1^ Published papers have shown that, when undergoing training and supervision, tracheostomized children aged 8 months and above had their speech and lung clearing improved by using a speaking valve, which they tolerated well.^18^

Valves make more spontaneous speech possible, without needing digital occlusion of the tracheostomy cannula. The psychological status of patients is improved, especially in terms of spontaneous speech and decreased tracheal secretions, and less productive coughing through the tracheostomy, all of which are important particularly in public.^19^

Use of a filter within the valve, which may be exchanged weekly, was done in the speaking valve we used in this study. It allows air entering the lower airways to carry in fewer pollutants. Less tracheostomy-associated aspiration has been observed when using speaking valves.^9^, ^13^, ^15^ A study in which videoendoscopy and videofluoroscopy were done in 16 patients with tracheostomies subdivided into two groups, one with and one without speaking valves, showed significant improvements in swallowing.^20^ There are studies, however, showing no influence of occlusion of the external orifice of the tracheostomy cannula on improved aspiration.^10^ Speaking valves yield benefits in the coughing reflex and improve lung hygiene in patients.

Available valves in the Brazilian market are imported and hard for patients to acquire in our milieu.^21^ The new speaking valve developed in this study is inexpensive; many patients may benefit from it and have their verbal communication and feeding in public improved.^19^

All patients in this study showed a marked improvement in qualify of life after beginning to use the speaking valve. Fourteen patients (43.75%) were able to use the valve full time, including during sleep. The other patients removed the valve for sleeping. The most adequate hardness for the speaking valve diaphragm was 40 and 50 Shores. Larger units increase the resistance of the diaphragm to opening when inhaling.

## CONCLUSION

The speaking valve was well adapted to all patients speaking without digital occlusion of the tracheostomy cannula.

A diaphragm hardness of 40 and 50 Shores should be used as standard in tracheostomized patients that are candidates for the speaking valve.
